# Umbelliferone Impedes Biofilm Formation and Virulence of Methicillin-Resistant *Staphylococcus epidermidis* via Impairment of Initial Attachment and Intercellular Adhesion

**DOI:** 10.3389/fcimb.2019.00357

**Published:** 2019-10-18

**Authors:** Thirukannamangai Krishnan Swetha, Murugesan Pooranachithra, Ganapathy Ashwinkumar Subramenium, Velayutham Divya, Krishnaswamy Balamurugan, Shunmugiah Karutha Pandian

**Affiliations:** Department of Biotechnology, Alagappa University, Karaikudi, India

**Keywords:** umbelliferone, biofilm, antibiotic resistance (AMR), methicillin-resistant *Staphylococcus epidermidis* (MRSE), antibiofilm, intercellular adhesion, initial attachment

## Abstract

*Staphylococcus epidermidis* is an opportunistic human pathogen, which is involved in numerous nosocomial and implant associated infections. Biofilm formation is one of the prime virulence factors of *S. epidermidis* that supports its colonization on biotic and abiotic surfaces. The global dissemination of three lineages of *S. epidermidis* superbugs highlights its clinical significance and the imperative need to combat its pathogenicity. Thus, in the current study, the antibiofilm activity of umbelliferone (UMB), a natural product of the coumarin family, was assessed against methicillin-resistant *S. epidermidis* (MRSE). UMB exhibited significant antibiofilm activity (83%) at 500 μg/ml concentration without growth alteration. Microscopic analysis corroborated the antibiofilm potential of UMB and unveiled its potential to impair intercellular adhesion, which was reflected in auto-aggregation and solid phase adherence assays. Furthermore, real time PCR analysis revealed the reduced expression of adhesion encoding genes (*icaD, atlE, aap, bhp, ebh, sdrG*, and *sdrF*). Down regulation of *agrA* and reduced production of secreted hydrolases upon UMB treatment were speculated to hinder invasive lifestyle of MRSE. Additionally, UMB hindered slime synthesis and biofilm matrix components, which were believed to augment antibiotic susceptibility. *In vivo* assays using *Caenorhabditis elegans* divulged the non-toxic nature of UMB and validated the antibiofilm, antivirulence, and antiadherence properties of UMB observed in *in vitro* assays. Thus, UMB impairs MRSE biofilm by turning down the initial attachment and intercellular adhesion. Altogether, the obtained results suggest the potent antibiofilm activity of UMB and the feasibility of using it in clinical settings for combating *S. epidermidis* infections.

## Introduction

*Staphylococcus epidermidis* is a Gram positive and coagulase negative commensal bacterium. It is a ubiquitous colonizer of skin, nostrils, head, armpit, and mucous membrane of healthy human and other mammals (Otto, 2009). This distinguished commensal can act as an opportunistic pathogen with high inclination toward immunosuppressed patients such as premature neonates, drug abusers, patients with indwelling medical devices, chronically hospitalized, and AIDS patients (Otto, 2009). It causes skin infections such as cellulitis, abscesses and several wound infections (Cogen et al., 2008). It also exacerbates the severity of miliaria or prickly heat by clogging the sweat pores *via* secretion of slimy extracellular polysaccharide substance (Mowad et al., 1995; Bukhari et al., 2016). It also triggers a spectrum of implant related infections (McCann et al., 2008) and several fatal systemic infections such as endocarditis and septicemia (Karchmer, 1985).

*S. epidermidis* exerts the aforesaid infectious facets by its ability to form biofilm on host tissues and medical implants (Le et al., 2018). Biofilm formation enables a single cell microorganism to presume transient multicellular behavior by forming multilayered aggregation of cells. This multifaceted biofilm is supported by extracellular polymeric matrix, which comprises proteins, lipids, carbohydrates, and DNA that altogether boosts its endurance to the stress posed by antibiotics and human innate defense system (Freitas et al., 2018). Additionally, the sturdiness of sessile cells to antibiotics is reported to be thousand times more than that of their planktonic counterpart (Cargill and Upton, 2009; de Oliveira et al., 2016). This conception is mainly believed to be the result of differential behavior of biofilm cells instigated by the differential gene expression and development of drug resistance due to Darwinian selection pressure (McCann et al., 2008; Mah, 2012). Further, the sequel of inappropriate intake of various antibiotics and changing trend of antibiotic resistant pattern of *S. epidermidis* open up the door to the prevalence of MDR *S. epidermidis* in clinical settings that still debilitates the effect of last alternative antibiotics namely, daptomycin, vancomycin, and linezolid (Fair and Tor, 2014; Barriere, 2015; Lee et al., 2018). Moreover, Lee et al. (2018) have reported the global dissemination of three MDR lineages of *S. epidermidis*, which highlights its clinical importance and triggers the inevitable need to probe an alternate medicine. To unwind these complications, the present study was designed to employ antivirulence therapy (Cegelski et al., 2008; Silva et al., 2016) wherein, the pathogenicity alone is perturbed without exerting any lethal effect on microbes. Also, the non-bactericidal nature of a drug is purported to reduce the probability of drug resistance and mutant development (Patsilinakos et al., 2019). In light of the aforesaid fact, several non-bactericidal antibiofilm molecules from natural sources have been reported recently against *S. epidermidis* (Artini et al., 2017; Ricciardelli et al., 2018; Patsilinakos et al., 2019).

In this context, plant derived compounds have been reported to extend vast structural diversity with ample beneficiary activities in handling infections (Saklani and Kutty, 2008). Umbelliferone (UMB), a coumarin derivative of benzopyrone is reported to be present in several plants and edible fruits (Ramalingam and Vaiyapuri, 2013). UMB exhibits broad spectrum pharmaceutical activities such as antihyperglycemic, antioxidant, antimicrobial, and antitumor activities (Mazimba, 2017). Albeit several prospective roles of UMB have already been reported, research study featuring the antibiofilm potential of UMB is scarce. Hence, the current study unravels the antibiofilm and antivirulence potential of UMB against infectious *S. epidermidis*, investigates the plausible mechanism of UMB using comparative gene expression study and validates the *in vitro* results using *Caenorhabditis elegans*, which is a simple *in vivo* model for toxicology and host-pathogen interaction studies.

## Materials and Methods

### Strains and Culture Conditions

In the present study, a methicillin-resistant and renowned biofilm positive (*ica* operon positive) strain, *S. epidermidis* ATCC 35984 (Cafiso et al., 2004) was used. It was maintained on tryptic soy agar (TSA) and cultured regularly in tryptic soy broth (TSB) at 37°C in a shaker incubator (160 rpm). The standard cell suspension of methicillin-resistant *S. epidermidis* (MRSE) for all *in vitro* experiments was prepared by adjusting the optical density (OD) of overnight culture to 0.4 at 600 nm (1 × 10^8^ CFU/ml) using TSB.

### Evaluation of Antibiofilm Effect of UMB

UMB (Catalog no. H24003-10G; Sigma Aldrich, Switzerland) was dissolved in methanol to prepare 10 mg/ml stock and refrigerated at 4°C until further use. The antibiofilm effect of UMB was studied using crystal violet (CV) staining method in 24-well polystyrene (hydrophobic) microtitre plate (Stepanović et al., 2007). Briefly, wells holding 1 ml of TSB with 1% inoculum (~1 × 10^6^ CFU/ml) was supplemented with increasing concentrations of UMB (100 to 500 μg/ml) and incubated for 24 h at 37°C in static condition. The untreated wells containing equal amount of vehicle (methanol) and wells containing TSB alone served as control and blank, respectively. The free-floating planktonic cells were discarded after incubation and loosely bound cells were removed by washing the wells twice with sterile distilled water. To quantify the biofilm biomass, 1 ml of CV stain (0.4% w/v) was added to the wells. After 10 min, wells were washed using water to remove surplus CV, air-dried and added with 1 ml of glacial acetic acid (10% v/v) for solubilizing the cell bound CV. The absorbance of eluted stain was spectrometrically measured at OD_570nm_, which indirectly relates the biofilm formation. The minimum biofilm inhibitory concentration (MBIC) of UMB was recorded as the minimum concentration, which displayed more than 80% of biofilm inhibition (Kwasny and Opperman, 2010). The antibiofilm effect of UMB was plotted as a percentage graph using the following formula.

Biofilm inhibition (%) = [(Control OD_570nm_ – Treated OD_570nm_) / Control OD_570nm_] ^*^ 100.

### Ring Biofilm Assay

The effect of UMB on ring biofilm formed at air-liquid interface was studied by growing MRSE in glass (hydrophilic) test tubes. Glass test tubes holding 2 ml of TSB and 1% inoculum (~1 × 10^6^ CFU/ml) was supplemented with increasing concentrations of UMB (100 to 500 μg/ml). Test tubes devoid of treatment were taken as control. The tubes were then incubated for 24 h in shaking condition (160 rpm) at 37°C. Biofilm formed on the glass tubes was stained with CV (0.4% w/v) as mentioned above to visualize the difference developed upon UMB treatment and photographed.

### Effect of UMB on MRSE Growth and Metabolic Viability

The effect of UMB on growth was studied using microbroth dilution assay in accordance with CLSI (2018). In brief, UMB was added at increasing concentrations (100–500 μg/ml) to the wells containing 1 ml of TSB and 1% inoculum (~1 × 10^6^ CFU/ml). The experimental condition was maintained similar to that of aforementioned biofilm study. After incubation, the absorbance was measured at OD_600nm_ using multilabel spectrophotometer (Spectramax M3, Molecular Devices, US). In continuation, the variation in metabolic activity of untreated and UMB treated cells was assessed using alamar blue (AB) assay as described by Pettit et al. (2005) with minor modifications. Precisely, cells (planktonic + biofilm) from each well were separately collected in fresh 2 ml tubes and harvested using centrifugation (10,000 rpm for 5 min), washed twice using sterile PBS (pH-7) and suspended in 1 ml of PBS. Then, 0.1 μM of resazurin or AB dye (Sigma Aldrich, Switzerland) was added to the tubes and incubated at 37°C for 2 h in dark condition. PBS comprising AB dye alone was considered as blank. Absorbance was read at OD_570nm_ and OD_600nm_ to calculate the percentage of reduction of resazurin (blue, oxidized form) to resorufin (fluorescent pink, reduced form) attributable to cellular metabolic reduction using the following formula

Reduction of AB dye (%) = [((E_oxi(OD1)_^*^T_OD1_) − (E_oxi(OD2)_^*^T_OD2_))/((E_red(OD1_)^*^B_OD1_) − (E_red(OD2)_^*^B_OD2_))]^*^100 wherein, E_oxi_–molar extinction coefficient of oxidized form of AB; E_red_–molar extinction coefficient of reduced form of AB; T—test samples; B—blank; OD1−570 nm; OD2−600 nm.

### Microscopic Analysis of Biofilm Architecture

The biofilm inhibitory potential of UMB was verified by employing microscopic techniques such as light microscope (LM), scanning electron microscope (SEM) and confocal laser scanning microscope (CLSM) as described by Viszwapriya et al. (2016).

#### LM Analysis

The sample preparation process for LM study included formation of MRSE biofilm in the absence and presence of UMB (at 500 μg/ml) on glass slides (1 × 1 cm) placed in 24-well polystyrene microtitre plate holding 1 ml of TSB and 1% inoculum (~1 × 10^6^ CFU/ml) for 24 h at 37°C. The glass slides were removed from the wells after incubation, washed thrice with sterile water to remove loosely bound or unbound cells and air-dried. Further, the glass slides were stained with CV (0.4% w/v) for 5 min, de-stained using sterile water to expel the surplus stain, air-dried and subsequently visualized and imaged at 400X magnification under light microscope (Nikon Eclipse 80i, USA).

#### SEM Analysis

For SEM observation, the biofilm in the absence and presence of UMB (at 500 μg/ml) was formed on glass slides as mentioned above. Further, the biofilms were fixed with glutaraldehyde (2.5% v/v) for 1 h, dehydrated using increasing concentrations of ethanol (20, 40, 60, 80, and 100% v/v) for 2 min and air-dried. The dried slides were then gold sputtered, visualized and imaged under SEM (VEGA 3 TESCAN, Czech Republic).

#### CLSM Analysis

The biofilm was allowed to form on glass and titanium (1 × 1 cm) pieces in the absence and presence of UMB (at 500 μg/ml) as described earlier. Then, the untreated and UMB treated biofilms formed on glass and titanium surfaces were stained with acridine orange (0.1% w/v) at dark condition for 5 min, de-stained, air-dried and imaged at 200X magnification under CLSM (LSM 710, Carl Zeiss, Germany). Further, Zeiss LSM Image Examiner and Zen 2009 image software (Carl Zeiss, Germany) were used for image processing and z-stack analysis, respectively. COMSTAT software (gifted by Dr. Claus Stenberg, Technical University of Denmark) was also employed to quantify biofilm entities such as biomass, maximum thickness, and surface to volume ratio for understanding the extent of antibiofilm effect of UMB over different surfaces.

### Effect of UMB on Aggregation and Adherence of MRSE

#### Auto-Aggregation Assay

Auto-aggregation is reported to be an important trait of *S. epidermidis*, which greatly induces the intercellular adhesion and upholds the stability of biofilm (Ziebuhr et al., 1997; Schaudinn et al., 2014). Thus aggregation rate of control and UMB treated cells was monitored by performing auto-aggregation assay as described by Kos et al. (2003) with certain modifications. Briefly, control and UMB (at 500 μg/ml) treated cells were harvested from 24 h grown culture by centrifugation (10,000 rpm for 10 min). The cells were suspended in 5 ml of sterile PBS and absorbance of 2 ml of cell suspensions was measured at OD_600nm_. Then, the tubes were incubated statically until all control cells aggregate at the bottom of the tube. The absorbance was measured at OD_600nm_ by carefully collecting 2 ml of cell suspension from top of each tube without any disturbance. The rate of aggregation is calculated using the following formula.

Rate of aggregation = (((OD_600nm(bi)_-OD_600nm(ai)_)/OD_600nm (bi)_)^*^100)

Wherein, bi—before incubation; ai—after incubation.

#### Rate of Bacterial Adherence to Polystyrene (Hydrophobic) Surface

To appraise the adherence rate of control and UMB treated cells to polystyrene (hydrophobic) surface, MRSE culture grown in the absence and presence of UMB (at 500 μg/ml) for 24 h was adjusted to 0.4 OD (1 × 10^8^ CFU/ml) initially. Then, 1 ml of control and treated cultures were added to the polystyrene microtitre wells separately. After 1 h of incubation, the non-adherent and loosely bound cells were discarded and the wells were thoroughly washed three times with sterile distilled water. The cells bound to the wells were completely scraped off, suspended in 1 ml of PBS, serially diluted and plated on TSA plates. After 24 h of incubation, the total number of CFU/ml was calculated to estimate the rate of adherence.

#### Rate of Bacterial Adherence to Type I Collagen

To investigate the adherence rate of control and UMB treated MRSE to type I collagen (Cn), a protocol suggested by Arrecubieta et al. (2007) was followed with some changes. Briefly, each microtitre well was coated with 50 μg/ml of type I Cn (Bicolor life science assays) prepared in PBS and incubated at 4°C overnight. Then, the wells were thoroughly washed thrice with sterile distilled water and non-adherent regions of the wells were blocked using bovine serum albumin (BSA; 2% w/v) for 1 h at 37°C. After blocking, the wells were again washed five times and added with 1 ml of 24 h grown control and UMB treated cultures (adjusted to 0.4 OD; 1 × 10^8^ CFU/ml), which was trailed by 1 h incubation. After incubation, the loosely bound cells were removed by washing the wells thrice. The adherent cells were collected by two consecutive incubations (30 s) with trypsin/EDTA (0.05%), washed, suspended in 1 ml PBS, serially diluted and spread plated on TSA plates. The number of colonies formed after 24 h of incubation was counted and CFU/ml was calculated to analyze the adherence efficiency of control and treated cells to type I Cn. The number of control and treated cells adhered to plain BSA (2% w/v) coated wells were subtracted from respective control and treated cells adhered to type I Cn coated wells, in order to obtain the actual number of cells adhered to type I Cn.

### Effect of UMB on Other Virulence Factors of MRSE

#### Slime Production

The impact of UMB on slime production was phenotypically assessed using Congo red agar (CRA) plate assay as mentioned by Freeman et al. (1989) with some modifications. Congo red (0.08%) was prepared in water, sterilized separately and added to TSA supplemented with sucrose (3.7% w/v) at 55°C. An aliquot of standard cell suspension was streaked on CRA plates in the absence and presence of UMB (at 500 μg/ml). The plates were then incubated at 37°C for 24 h, visually observed for difference and photographed. Blackness of colonies was taken as the representation of slime synthesis, whereas absence or decreased blackness represented reduction in slime synthesis.

#### Protease Production

Protease production of MRSE was qualitatively estimated using caseinase assay as described by Liu et al. (1996) with modifications. Briefly, TSA plates containing 1% casein as substrate were prepared in the absence and presence of UMB (at 500 μg/ml). One microliter of standard cell suspension was spot inoculated at the center of the agar plates. The plates were then incubated at 37°C for 48 h and observed for white opaque zone around the bacterial colony. The plates were documented using high resolution charge-coupled device (CCD) camera (GelDoc XR+, Bio-Rad) and the zone diameter of proteolysis was measured using HiAntibiotic zone scale (Hi-Media Laboratories, India).

#### Lipase Production

The lipolytic activity of MRSE grown in the absence and presence of UMB (at 500 μg/ml) was quantified by performing lipase assay as described by Gupta et al. (2002) with some changes. The culture supernatant of 24 h grown control and treated samples was collected by centrifugation (10,000 rpm for 10 min). To 100 μl of supernatant, 900 μl of substrate mixture containing one volume of 0.3% p-nitrophenyl palmitate in 2-propanol and nine volumes of 0.2% (w/v) sodium deoxycholate and 0.1% (w/v) gummi arabicum in 50 mM Na_2_PO_4_ buffer (pH-8) was added. The reaction mixture was then incubated at dark condition for 1 h. The reaction mixtures were centrifuged (12,000 rpm for 5 min), which was followed by addition of 1 ml of 1 M Na_2_CO_3_ to the supernatant to stop the reaction. Then, the absorbance was measured at OD_410nm_ for quantifying the lipase production.

### Estimation of Biofilm Components in the Absence and Presence of UMB

To estimate the components associated with biofilm, MRSE was grown in the absence and presence of UMB (at 500 μg/ml) in 6 well polystyrene microtitre plate at 37°C for 24 h. After incubation, the planktonic cells were discarded without disturbing the biofilm and loosely bound cells were removed by washing the wells thrice with sterile distilled water. Then biofilm cells were scraped off from the wells, collected in fresh tubes using 200 μl of TE buffer (10 mM Tris and 10 mM EDTA; pH-8) and processed accordingly.

#### Carbohydrates

The total carbohydrate content of biofilm cells was estimated using phenol-sulfuric acid method described by Dubois et al. (1956) with some changes. Briefly, biofilm cells in 200 μl of TE buffer were added with equal volume of 5% phenol followed by addition of 5 volumes of concentrated sulfuric acid containing 0.2% of hydrazine sulfate. The mixture was then incubated for 1 h at dark condition and spectrometrically read at OD_490 nm_.

#### Lipids

The total lipid estimation of biofilm cells was carried out using quick colorimetric method adapting sulpho-phospho-vanillin reaction with modifications (Byreddy et al., 2016). Briefly, 100 μl of biofilm cells was added to 200 μl of sulfuric acid and incubated at room temperature for 10 min followed by incubation on ice for 5 min. Then 5 ml of phosphovanillin reagent (0.12% of vanillin dissolved in 20 ml of hot water and made up to 100 ml using phosphoric acid) was added to the cells, incubated at 37°C in shaking condition for 15 min and absorbance was measured at OD_530 nm_.

#### Proteins and Extracellular DNA (eDNA)

Briefly, 200 μl of biofilm cells was vortexed for 5 min to extricate the biofilm matrix, centrifuged (12,000 rpm for 5 min) and supernatant (containing biofilm matrix associated proteins and eDNA) was collected to assess the protein and eDNA content. Two hundred microliter of control and treated supernatants were taken for protein estimation by Bradford method. While, equal volume of control and treated supernatants was taken for visually appraising the eDNA content of biofilm matrix by performing agarose gel electrophoresis (AGE) with 1.2% agarose gel and documented using high resolution CCD camera (GelDoc XR+, Bio-Rad).

### Extraction and Fourier Transform Infrared (FTIR) Analysis of Extracellular Polymeric Substances (EPS)

The EPS from control and UMB treated cells was extracted using the procedure suggested by Badireddy et al. (2008) with certain changes. Briefly, the cell pellet and cell free culture supernatant (CFCS) of 24 h culture grown in the absence and presence of UMB (at 500 μg/ml) were separated by centrifugation (10,000 rpm for 10 min). The cell pellets were washed using PBS, suspended in isotonic buffer (10 mM Tris-HCl pH-8, 10 mM EDTA and 2.5% NaCl) and incubated overnight at 4°C. Consequently, the cell suspensions were vortexed for 5 min and centrifuged (10,000 rpm for 15 min at 4°C) to collect cell-bound EPS present in the supernatants. Cell-bound EPS was then pooled with CFCS containing cell-free EPS, which was followed by the addition of three volumes of ice cold ethanol and subsequent incubation at −20°C overnight to precipitate EPS. After incubation, the EPS was separated using centrifugation (8,000 rpm for 20 min at 4°C), washed with 70% ethanol and dried using vacuum drier (Christ Alpha 2-4 LD plus, UK). The extracted EPS was then mixed with potassium bromide (KBr) in the ratio 1: 100 and compressed into pellet using manual hydraulic press. Spectral scan of control and treated EPS was done in the range of 4,000–400 cm^−1^ with the resolution of 4 cm^−1^ using FTIR spectrophotometer (Nicolet iS5, Thermo Fisher Scientific Inc., USA). The spectrum of KBr was also taken and nullified from all other spectra.

### Antibiotic Susceptibility Testing (AST)

Initially, the minimum inhibitory concentration (MIC) of test antibiotics was assessed using microbroth dilution assay (CLSI, 2018) as described earlier. MIC is determined as minimum concentration that displayed complete visible growth inhibition of MRSE following 24 h of incubation. Then, the influence of UMB on MIC of test antibiotics was examined by Kirby–Bauer agar diffusion method. Preliminarily, the stock solutions of gentamycin, rifampicin, vancomycin, and linezolid (Hi-Media Laboratories, India) were prepared. Then, overnight culture adjusted to 0.5 McFarland was swabbed uniformly on TSA plates supplemented with and without UMB (at 500 μg/ml) using sterile cotton swabs. Further, wells of 3 mm diameter were punched at the center of the agar plates and test antibiotics at their respective MIC were added to the wells. In parallel, UMB at 500 μg/ml was also loaded in the well of a plain TSA plate, in order to evaluate its individual antibacterial effect. After incubation for 24 h at 37°C, the zone diameter of antibacterial activity in control and treated plates was measured using HiAntibiotic zone scale (Hi-Media Laboratories, India) and subsequently, the plates were documented using high resolution CCD camera (GelDoc XR+, Bio-Rad).

### Gene Expression Study Using Real Time PCR

To investigate the effect of UMB on gene expression pattern, total RNA from mid-log phase of control and UMB (at 500 μg/ml) treated cultures was isolated using the protocol described by Oh and So (2003). Further, cDNA synthesis from isolated mRNA transcripts was done using High Capacity cDNA Reverse Transcription Kit (Applied Biosystems, USA). The synthesized cDNA was then added to biofilm associated gene-specific primers (*agrA, icaA, icaD, aap, bhp, ebh, atlE, sdrG, sdrH*, and *sdrF*) and PCR mix (SYBR Green Kit, Applied Biosystems, USA) at a predefined ratio. The primers used in this study were designed with the help of Primer3 software and synthesized by Sigma Aldrich, Switzerland. The details of primer sequences and specific roles of candidate genes are given in Table 1. Housekeeping gene, *rplU* (50S ribosomal protein) was considered as the internal control. The thermal profiling for real time PCR analysis included initial denaturation at 95°C for 5 min trailed by 30 cycles of denaturation at 95°C for 1 min, annealing at 58°C for 1 min, extension at 72°C for 1 min and final extension at 72°C for 5 min. The cycle threshold (Ct) values of candidate genes obtained from quantitative PCR analysis were normalized with Ct value of *rplU* (ΔCt) and differential gene expression pattern was calculated using comparative threshold method (ΔΔCt) (Livak and Schmittgen, 2001).

**Table 1:** Primer sequences and function of candidate genes used in the study.

**S. No**.	**Primer**	**Sequence (5–3^**′**^)**	**Function**	**References**
1.	*rplU*-F	TTGTAGGTGGCGACTCAGTT	Housekeeping gene encoding 50S ribosomal protein	Kannappan et al., 2019
	*rplU*-R	ATGGTTGACGATGGCCTTTT		
2.	*agrA*-F	TGTAACCAGTCACAGTGAGCT	Encodes response regulator of agr quorum sensing system	This study
	*agrA*-R	CCCCGCTTTAACTCAATCGT		
3.	*icaA*-F	TTGATGACGATGCGCCTTTT	Encodes N-acetyl glucosaminyl transferase essential for PIA synthesis	Sivaranjani et al., 2019
	*icaA*-R	CTGCAAGAGATTGACTTCGCT		
4.	*icaD*-F	GACAGAGGCAATATCCAACGG	Encodes N-acetyl glucosaminyl transferase crucial for complete transferase activity of icaA and synthesis of functional PIA	This study
	*icaD*-R	ACAAACAAACTCATCCATCCGA		
5.	*aap*-F	GGGCAAACGTAGACAAGGTC	Encodes accumulation associated protein essential for biofilm formation	Kannappan et al., 2019
	*aap*-R	GCTTTCGCTTCATGGCTACT		
6.	*bhp*-F	TGATGACAACGCAACGACAA	Encodes cell wall associated accumulation protein required for biofilm formation	Kannappan et al., 2019
	*bhp*-R	TGGTGTTGGACTCGTAGCTT		
7.	*ebh*-F	CTAAAGGAACATGGGCAGGC	Encodes cell wall associated fibronectin binding protein	Kannappan et al., 2019
	*ebh*-R	AAACACCCCAGTTGCTAGGA		
8.	*atlE*-F	ATAGAAACGGTGTGGGACGT	Encodes autolysin that supports biofilm formation through autolysis mediated eDNA release. Also encodes adhesin that facilitates attachment of cells to polystyrene surfaces, vibronectin, fibrinogen and fibronectin (matrix proteins)	Kannappan et al., 2019
	*atlE*-R	ACCTGCACCCCAAGATAAGT		
9.	*sdrG*-F	GTGACTTGCCTCCTGAAAAA	Encodes serine aspartate repeat protein that binds fibrinogen	Kannappan et al., 2019
	*sdrG*-R	TCCGGTGTTTCGAATGTAAT		
10.	*sdrF*-F	TGAAAAAGAGAAGACAAGAACCA	Encodes serine aspartate repeat protein that binds collagen	Kannappan et al., 2019
	*sdrF*-R	GATTGTCTTCAGCCGCTTTA		
11.	*sdrH*-F	AAAAAGCCATTTTTGTTCCA	Encodes serine aspartate repeat protein that aids uncharacterized binding	This study
	*sdrH*-R	CATACGAATCAACCCCAAAG		

### Evaluation of Toxicity and *in vivo* Efficacy of UMB Using *Caenorhabditis elegans*

The eukaryotic miniature model, *C. elegans* was used for the examination of cytotoxicity of UMB and validation of antibiofilm, antivirulence and anti-adherent properties of UMB against MRSE under *in vivo* condition. *C. elegans* maintenance was performed in accordance with the standard protocol described earlier (Brenner, 1974). *Escherichia coli* OP50 (1 × 10^6^ cells/ml) was given as laboratory food source to *C. elegans*. For cytotoxicity analysis, *C. elegans* liquid survival assay was carried out as described by Srinivasan et al. (2018) with slight changes. Briefly, quantifiable number of hermaphrodites (~10) at L4 stage was taken in 1 ml of M9 buffer (0.3% KH_2_PO_4_, 0.6% Na_2_HPO_4_, 0.5% NaCl, and 0.1 ml 1 M MgSO_4_) supplemented with and without UMB (at 500 μg/ml) and their survival rate was monitored at regular intervals for 96 h.

Further, the efficacy of UMB on the survivability of infected worms was inspected using two groups of worms (~20 numbers each) infected with MRSE wherein, one grown in the presence of methanol (control) and the other was grown in the presence of UMB (at 500 μg/ml). The survivability of worms was then monitored at regular intervals for 96 h. To estimate the degree of *S. epidermidis* infection *in vivo* using CFU assay, two groups of worms grown in the presence of MRSE + methanol (control) and MRSE+ UMB (treated) were prepared. Then, the bacterial load in control and treated groups was assessed using CFU assay as described by Gowri et al. (2018) with modifications. Briefly, worms were washed after 12 h of bacterial exposure to discard non-adherent bacteria. Then worms were taken in 100 μl of PBS and crushed using mini-homogenizer (Moxcare Labware, MT-13K) to extract MRSE. Further, the extracted bacteria were serially diluted and plated on TSA plates. After 24 h of incubation, the bacterial load in control and treated groups was enumerated by manual colony counting and CFU/ml calculation.

To assess the influence of UMB on adherence and biofilm formation of MRSE on *C. elegans* cuticle (Cn rich layer), an aliquot of 24 h culture grown in the absence and presence of UMB (at 500 μg/ml) was seeded separately on nematode growth medium (NGM) agar plates and quantifiable number of L4 stage worms (~20) was transferred to the NGM plates aseptically. Then, the plates were incubated at 20°C for 5 days. After incubation, the worms were washed with M9 buffer, anesthetized using 1 mM sodium azide and then monitored under light microscope for biofilm formation. The worms grown in *E. coli* OP50 seeded NGM plate was taken as the standard for appraising adherence and biofilm formation in infected and treated worms.

### Statistics

All experiments were performed in at least three biological and two experimental replicates. The data were represented as mean value ± standard deviation. The statistical significance between control and treated samples was examined with Student's *T*-test and one way ANOVA trailed by Dunnett's test using SPSS (Chicago, IL, USA) software package.

## Results

### Non-bactericidal Antibiofilm Effect of UMB

**Figure 1:**
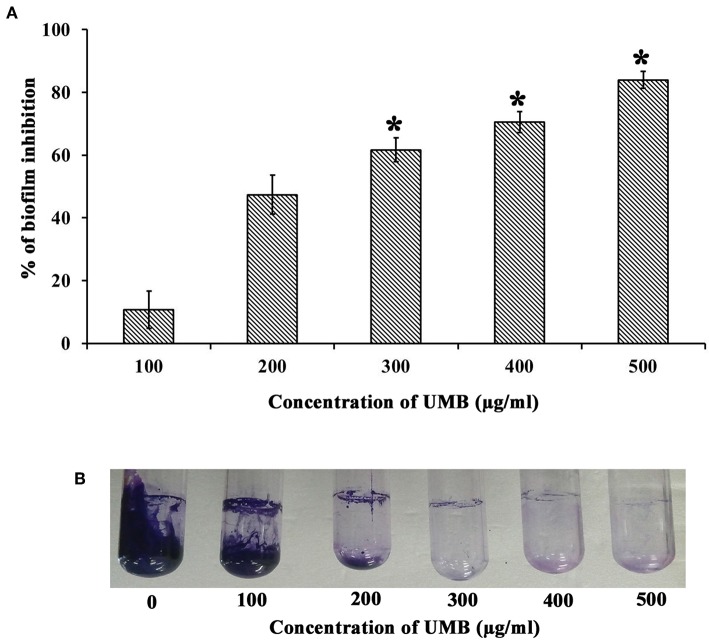
Effect of UMB on biofilm formation of MRSE. **(A)** Bar graph represents dose dependent inhibition of MRSE biofilm upon treatment with UMB. UMB exhibited a maximum of 83% biofilm inhibition of MRSE at 500 μg/ml concentration, which was fixed as MBIC. **(B)** Representative images of glass tubes showcasing the CV stained ring biofilm of MRSE formed in the absence and presence of UMB at varying concentrations. Error bars represent standard deviations. *Statistical significance between control and treated samples from three independent experiments (*p* < 0.005).

**Figure 2:**
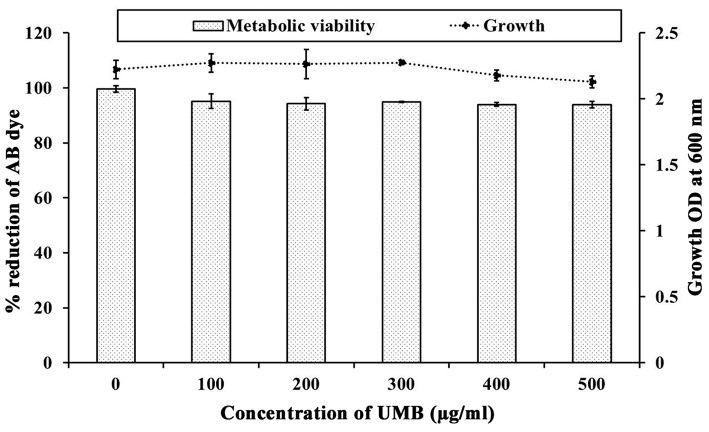
Influence of UMB on growth and metabolic activity of MRSE. Line graph indicates the effect of UMB on MRSE growth assessed using microbroth dilution assay. Bar graph denotes the effect of UMB on metabolic activity of MRSE tested using alamar blue assay. Error bars indicate standard deviations.

The CV staining method showed the dose dependent antibiofilm activity of UMB with a maximum of 83% biofilm inhibition at 500 μg/ml concentration (Figure 1A). UMB also efficiently reduced the ring biofilm formation of MRSE in a dose dependent manner, which was verified by visual observation of CV stained control and treated ring biofilms formed on glass test tubes (Figure 1B). Thus, MBIC of UMB was fixed as 500 μg/ml and used to carry out all bioassays. The microbroth dilution assay revealed the non-bactericidal nature of UMB at all tested concentrations. Further, the metabolic activity test divulged unaltered metabolic activity of control and UMB treated cells (Figure 2).

### Microscopic Revelation of Abridged Biofilm Formation Upon UMB Treatment

**Figure 3:**
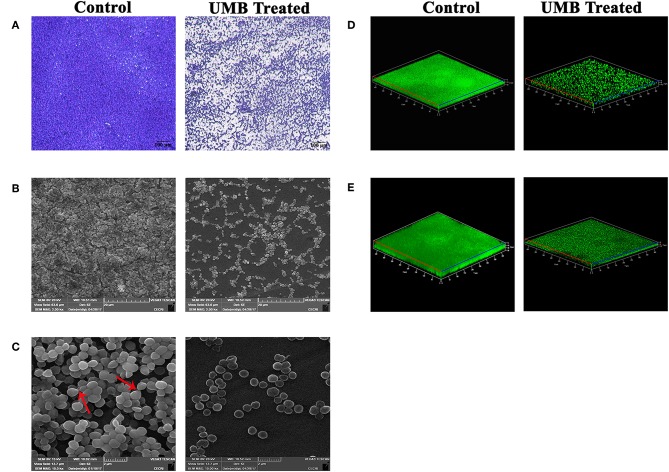
Microscopic images of MRSE biofilm formed in the absence and presence of UMB (at 500 μg/ml). **(A)** LM (400X, scale bar-−100 μm), **(B)** SEM (2000X, scale bar-−20 μm), and **(C)** SEM (10,000X, scale bar-−2 μm) images displaying reduced biofilm surface coverage area and less aggregated monolayer biofilm in UMB treated glass slides than that of control glass slides. Red arrows in SEM image of control slide (10,000X) denote adhesiveness between cells. Adhesiveness could not be seen in treated slide. Three dimensional CLSM images depicting significant reduction of biofilm formation on **(D)** glass as well as **(E)** titanium surfaces upon treatment with UMB (at 500 μg/ml).

LM and SEM observations revealed the presence of less aggregated monolayered biofilm in UMB treated slides (Figures 3A–C). Whereas, untreated biofilm was found to be multilayered and highly aggregated with high surface coverage area. SEM images captured at higher magnification confirmed the presence of scattered biofilm with very meager adhesion between the cells upon UMB treatment. However, control SEM image showcased three-dimensional robust biofilm with cells clumped together by adhesive nature. The CLSM visualization of biofilm formed on glass and titanium surfaces clearly portrayed the potency of UMB which considerably reduced adherence of MRSE to various substrata (Figures 3D,E). The quantification of biofilm entities using COMSTAT software validated the antibiofilm effect of UMB by divulging reduced biomass and thickness with increased surface to volume ratio in UMB treated samples (Table 2).

**Table 2:** COMSTAT analysis of MRSE biofilm formed on glass and titanium surfaces in the absence and presence of UMB (at 500 μg/ml).

**S. No**.	**Parameter**	**Glass**		**Titanium**	
		**Control**	**Treated**	**Control**	**Treated**
1.	Biofilm biomass (μm^3^/μm^2^)	24.99	21	31.92	21.525
2.	Maximum thickness (μm)	23.8	20	30.4	20.5
3.	Surface to volume ratio (μm^2^/μm^3^)	0.044725	0.05232	0.035585	0.049385

### UMB Treatment Mitigates the Rate of Bacterial Aggregation and Adherence

**Figure 4:**
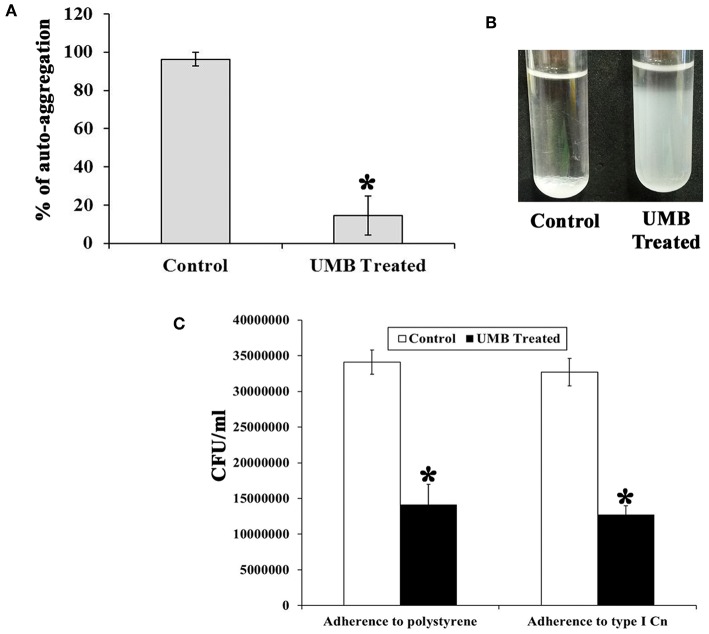
Effect of UMB on rate of aggregation and adherence. **(A)** Bar graph depicts the rate of aggregation of MRSE cells in the absence and presence of UMB (at 500 μg/ml). **(B)** Representative image of test tubes displaying abridged aggregation of UMB treated MRSE than that of the control cells. **(C)** Bar graph shows the rate of adherence of MRSE in the absence and presence of UMB (at 500 μg/ml) indicating the reduced adherence of MRSE to polystyrene and type I Cn coated surfaces by UMB. Error bars indicate standard deviations. *Statistical significance between control and treated samples from three independent experiments (*p* < 0.005).

The untreated control cells took ~2 h 30 min to completely aggregate. Whereas, UMB treated cells did not aggregate completely even after 12 h of incubation. The aggregation rate of UMB treated cells was observed to be drastically reduced up to 88% when compared to that of untreated control (Figures 4A,B).

Additionally, the bacterial adherence to polystyrene (hydrophobic) surface was studied wherein, the adherence rate of UMB treated cells to polystyrene surface was found to be reduced up to 58% than control cells, which implies the antiadherence role of UMB. Furthermore, the bacterial adherence to type I Cn was also assessed. Interestingly, the adherence rate of UMB treated cells to Cn was found to be decreased up to 60% when compared to that of control (Figure 4C).

### UMB Reduces the Production of Slime and Secreted Hydrolases and Diminishes the Content of Biofilm Components

**Figure 5:**
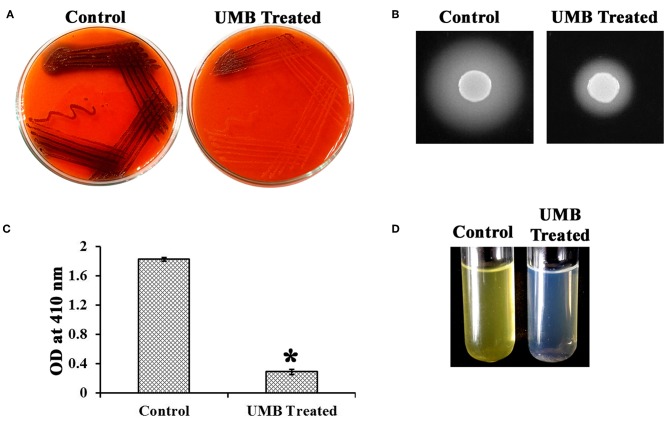
Effect of UMB on production of slime and secreted hydrolases. **(A)** CRA assay indicating the reduced slime production upon UMB treatment. **(B)** Caseinase assay depicting the reduced zone of protease production upon UMB treatment. **(C)** Bar graph depicting the reduction in lipase production of MRSE upon UMB treatment. **(D)** Representative image of test tubes showcasing the reduced yellow color development in UMB treated tube than that of control, implying the reduction in lipase production upon UMB treatment. *Statistical significance between control and treated samples from three independent experiments (*p* < 0.005).

**Figure 6:**
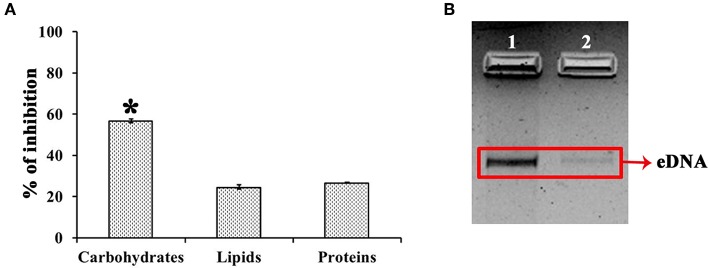
Effect of UMB on biopolymers of biofilm matrix. **(A)** Bar chart depicting the percentage inhibition of carbohydrates, lipids and proteins of biofilm matrix upon UMB treatment. **(B)** Agarose gel image indicating the effect of UMB on eDNA content of biofilm matrix. Lane 1—MRSE control; Lane 2—MRSE treated with UMB (at 500 μg/ml). The red box highlights the eDNA band of control and UMB treated samples. *Statistical significance between control and treated samples from three independent experiments (*p* < 0.005).

UMB treatment was found to diminish the blackness of colonies when compared to that of control, which clearly indicates the potential of UMB to reduce slime production (Figure 5A). Caseinase assay divulged the reduced zone of proteolysis in UMB treated plate (16 mm) when compared to control plate (25 mm) (Figure 5B). Also, the lipase production was found to be reduced up to 60% upon UMB treatment (Figures 5C,D). As UMB potentially inhibited biofilm formation, its effect on biopolymers of extracellular matrix (ECM) was also quantified. UMB treatment was found to reduce carbohydrate, lipid, and protein content of biofilm up to 57, 26, and 27% respectively (Figure 6A). Additionally, the eDNA content of UMB treated biofilm was found to be greatly reduced than control as witnessed using AGE (Figure 6B).

### FTIR Analysis of Untreated and UMB Treated EPS

**Figure 7:**
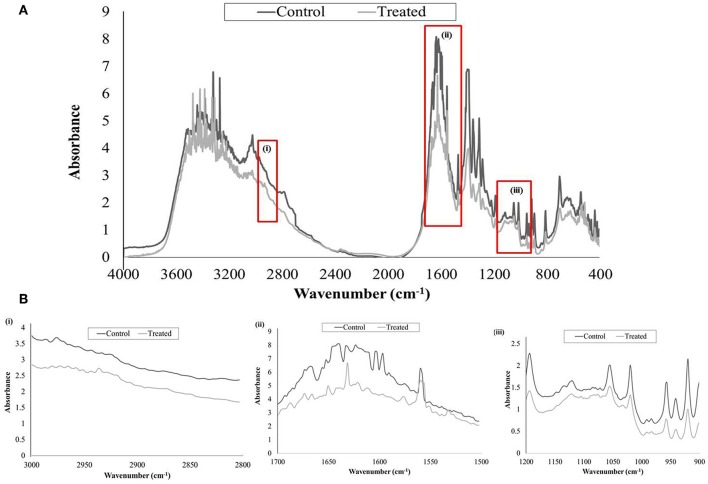
FTIR analysis of untreated and UMB (at 500 μg/ml) treated MRSE, EPS. **(A)** Spectra of control and UMB treated MRSE, EPS scanned in the range of 4,000–400 cm^−1^, specifying the regions corresponding to (i) lipids (3,000–2,800 cm^−1^); (ii) amide bonds of proteins & peptides (1,700–1,500 cm^−1^); and (iii) polysaccharides (1,200–900 cm^−1^). **(B)** Enlarged images of highlighted spectral regions (i), (ii), and (iii).

FTIR analysis was done to study the alterations in EPS of control and UMB treated samples by covering spectral range 4,000–400 cm^−1^, wherein the three highlighted regions *viz.*, 3,000–2,800, 1,700–1,500, and 1,200–900 cm^−1^ represented the absorption of lipids, amide linkages of proteins & peptides and mixed polysaccharides & nucleic acids, respectively. The overall absorption of UMB treated EPS was found to be reduced than untreated control with considerable modification in the spectral pattern of the region, 1,700–1,500 cm^−1^ (Figure 7).

### Augmented Antibiotic Susceptibility of MRSE Upon UMB Treatment

Since UMB effectively inhibits biofilm formation, its influence on activity of conventional antibiotics was assessed using Kirby–Bauer test. Increase in zone diameter of UMB treated plates with respect to the control plates clearly affirmed the potential of UMB to promote the antibacterial activity of conventional antibiotics. No zone of growth inhibition was observed in agar plates loaded with UMB alone, which implied the non-bactericidal effect. The zone diameter of antibacterial activity observed in control and UMB treated plates is given in Table 3.

**Table 3:** Zone of antibiotic susceptibility of MRSE in the absence and presence of UMB (at 500 μg/ml).

**S. No**.	**Antibiotic**	**Concentration of antibiotic (μg/ml)**	**Zone diameter in control plate (mm)**	**Zone diameter in treated plate (with 500 μg/ml of UMB) (mm)**
1.	Gentamycin	120	12	15
2.	Rifampicin	0.5	22	24.5
3.	Vancomycin	10	14	17
4.	Linezolid	250	12	16.5

### Differential Gene Expression Analysis

**Figure 8:**
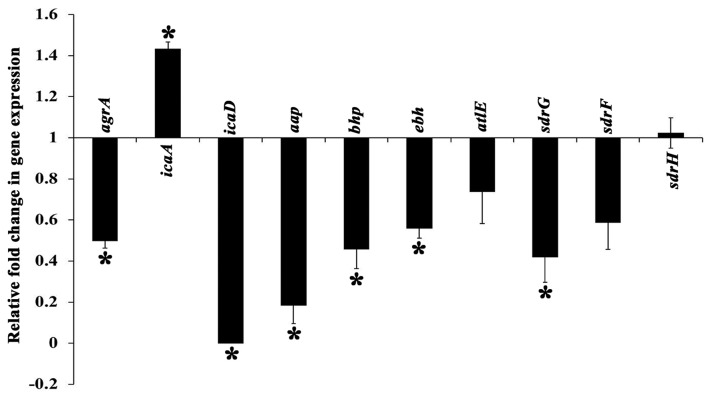
Differential gene expression profile of MRSE in the absence and presence of UMB (at 500 μg/ml). The expression profile of candidate genes entailed in biofilm formation and virulence factor production was found to be down regulated upon UMB treatment. The housekeeping gene, *rplU* was considered as the internal control. Error bars represent standard deviations. *Statistical significance between control and treated samples from three independent experiments (*p* < 0.005).

The expression profile of genes involved in the regulation of MRSE biofilm formation was analyzed using real time PCR (qPCR), which encompassed genes encoding exopolysaccharride (PIA) synthesis (*icaA* and *icaD*), virulence & biofilm formation (*agrA*), intercellular adhesion & accumulation (*aap* and *bhp*), autolysin/adhesin (*atlE*) and ECM binding protein (*ebh, sdrH, sdrG*, and *sdrF*). The expression of candidate genes in the absence and presence of UMB was compared, which revealed the down regulation of genes such as *agr A, icaD, aap, bhp, ebh, atl E, sdrG*, and *sdrF* upon UMB treatment, attesting the biofilm inhibitory potential of UMB. On the other hand, UMB treatment was found to up regulate the expression of *ica A* and insignificantly affect the expression of *sdrH* (Figure 8).

### *In vivo* Antibiofilm and Antiadherence Property of Non-toxic UMB Against MRSE

**Figure 9:**
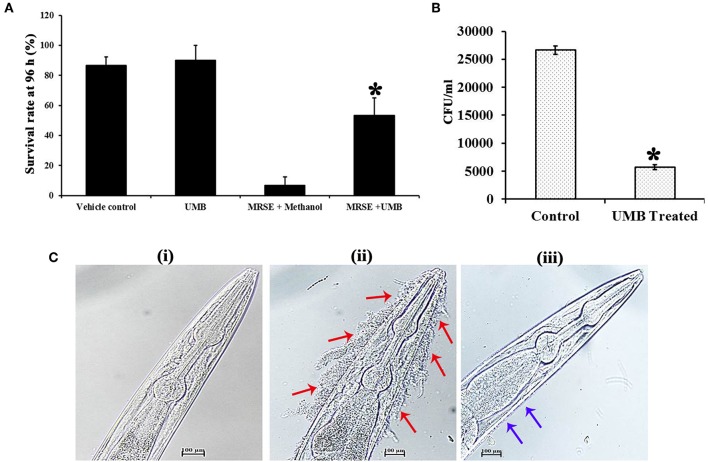
Evaluation of cytotoxicity and *in vivo* efficacy of UMB using nematode model *C. elegans*. **(A)** The survivability of *E. coli* OP50 fed *C. elegans* was increased upon UMB treatment, attesting the innocuous nature as well as health benefit of UMB. The increased survivability of MRSE infected worms in the presence of UMB signifies the antivirulence activity of UMB. **(B)** Bar chart depicting the decreased bacterial load in UMB treated worms than that of untreated control worms, confirming the antibiofilm efficacy of UMB. **(C)** LM images (400X, scale bar-−100 μm) of anterior part of *C. elegans* grown in the presence of (i) *E. coli* OP50, (ii) MRSE, and (iii) MRSE + UMB (at 500 μg/ml). The adherence of UMB treated MRSE (indicated by blue arrows) to *C. elegans* cuticle is considerably reduced than untreated MRSE (indicated by red arrows), endorsing the antiadherence property of UMB. Error bars depict standard deviation. *Statistical significance between control and treated samples from three independent experiments (*p* < 0.005).

The survivability of worms in the absence and presence of UMB was monitored for 96 h. Strikingly, the survivability of worms fed with UMB + *E. coli* OP50 was found to be slightly higher than control worms fed with *E. coli* OP50 alone, which confirms the non-toxic nature of UMB as well as unveils its capability to extend additional benefit of embellishing the survivability rate. Similarly, the potency of UMB to restore the survivability of infected worms was also evaluated for 96 h, which divulged the enhanced survival rate of treated group (worms + MRSE + UMB) than that of control group (worms + MRSE + methanol) (Figure 9A). Further, CFU assay corroborated the antivirulence efficiency of UMB, wherein bacterial load in *C. elegans* was found to be reduced upon UMB treatment (Figure 9B).

Furthermore, the adherence and subsequent biofilm formation of UMB treated MRSE on the cuticle of *C. elegans* was found to be less when compared to untreated MRSE, which efficiently adhered to anterior part of the worm and formed thick multilayered biofilm. On the other hand, no adherence or biofilm formation was observed in standard group (Figure 9C). Altogether, the *in vivo* assays using *C. elegans* demonstrated the non-toxic nature, antibiofilm, antivirulence and anti-adherent properties of UMB.

## Discussion

Biofilm formation of *S. epidermidis* on animate (host tissues) and inanimate (medical implants) substances is reported to govern hostile events including the emergence of antibiotic resistance, host system weakening, escalation of morbidity and mortality rates (Izano et al., 2008; Otto, 2009; Fey and Olson, 2010). Moreover, the dissemination of three MDR lineages of *S. epidermidis* across 24 countries (Lee et al., 2018) has marked the importance of exploration of alternate medicine to tackle their pathogenicity. In this backdrop, plant sources are reported to serve quintessential roles in medical field (Saklani and Kutty, 2008). Thus, this study delved the antagonistic effect of umbelliferone (UMB), a phytocompound against *S. epidermidis* biofilm and virulence. The presence of UMB in many plants (cumbungi, giant fennel, garden angelica, chamomile, cinnamon etc.) and edible sources (carrot, coriander, asafoetida, bitter orange, grapefruit, Bengal quince etc.) together with its versatile medicinal properties (Mazimba, 2017) highlight the context of utilizing UMB in the present study.

UMB was preliminarily tested against biofilm formation of MRSE in polystyrene (hydrophobic) and glass (hydrophilic) surfaces, wherein UMB exhibited dose dependent antibiofilm activity with maximum inhibition at 500 μg/ml concentration (Figures 1A,B). This confirms the surface independent antibiofilm efficacy of UMB. Further, microbroth dilution and AB assays showed the non-bactericidal nature of UMB (Figure 2). This non-lethal effect of UMB on MRSE is expected to have reduced probabilities of drug resistance development, since killing effect of a drug is stated to aid in drug resistance by triggering Darwinian selection pressure on microbes (McCann et al., 2008; Dharmaprakash et al., 2015). With these observations, 500 μg/ml of UMB was fixed as MBIC and used for subsequent bioassays.

Additionally, microscopic analysis validated the antibiofilm potential of UMB wherein, UMB hindered initial attachment and adhesiveness between cells (Figures 3A–D). Adhesiveness is speculated as intercellular adhesion that is nurtured by adhesive molecules such as PIA, Aap, or Embp during accumulative phase of biofilm (Mack et al., 1996; Hussain et al., 1997; Christner et al., 2010). Thus, microscopic observation hints the potential of UMB to hamper production of adhesive molecules that are essential for building a robust biofilm consortium.

As titanium and its alloys are prevalently used biomaterials for implantation (Albrektsson et al., 1983; Wisbey et al., 1991), the antibiofilm efficacy of UMB on titanium surfaces was assessed by CLSM which showcased the antibiofilm effect of UMB on titanium surfaces (Figure 3E and Table 2). This surface independent activity of UMB underlines the feasibilities of employing UMB in clinical settings for impeding biofilm formation of MRSE on UMB coated implants.

Apart from this, adherence and aggregation are regarded as crucial phenotypes of biofilm positive *S. epidermidis*, which supports bacterial attachment to diverse substrata, intercellular adhesion, regulation of cell integrity in bacterial aggregates and evasion from host defense (Ziebuhr et al., 1997; Vuong et al., 2004; Rohde et al., 2005). Besides, aggregation is also linked with the expression of adhesive molecules (Fey and Olson, 2010). Auto-aggregation assay elucidated the potency of UMB to delay the rate of aggregation (Figures 4A,B), which indirectly suggests the role of UMB in reducing adhesive molecules and biofilm formation. Additionally, solid phase adherence assay portrayed the adjourned adherence rate and reduced bacterial attachment to polystyrene and type I Cn coated surfaces upon UMB treatment (Figure 4C). The low affinity of UMB treated cells to polystyrene surface highlights the potential of UMB to reduce primary attachment and subsequent colonization. Further, the ability of *S. epidermidis* to adhere to ECM/plasma proteins that cover biomaterials shortly after implantation has greatly affected the implant infection rate (Herman et al., 2013). Thus, the reduced adherence of UMB treated *S. epidermidis* to Cn (ECM protein) coated surface relates the efficacy of UMB to reduce binding of *S. epidermidis* to host proteins and subsequent implant infection rate.

Biofilm positive phenotype is also linked with slime synthesis, which results in blackness of colony on CRA plate (Arciola et al., 2006). CRA assay revealed significant reduction in blackness of colony upon UMB treatment (Figure 5A), which provides an added detail to the antibiofilm potential of UMB. Clinically, the invasive nature of *S. epidermidis* is promoted by secreted hydrolases production, which supports skin colonization, destruction of signal peptides, host invasion, and elusion from antibiotic treatment and host defense system (von Eiff et al., 2002; Michelim et al., 2005). UMB was effective in impeding protease and lipase production (Figures 5B–D). In connection with this, Vuong et al. (2000) have stated the production of proteases and lipases to be other vital pathogenic determinants of *S. epidermidis*, which was found to be reduced in agr mutant they have constructed. Additionally, Sethupathy et al. (2017) have reported the protease and lipase inhibitory potential of L-ascorbyl 2,6-dipalmitate in *Staphylococcus aureus*. Besides, exoproteome modulation of bone model by *S. aureus* protease has been recounted to be attributed to its enhanced virulence during invasive infections (Cassat et al., 2013). Accordingly, the inhibitory potential of UMB on secreted hydrolases production observed in this study is envisaged to deter progress of *S. epidermidis* infection to invasive stage.

The EPS matrix embedding the sessile cells is described to escalate and sustain the resistivity of *S. epidermidis* to various environmental stresses (Schaeffer et al., 2016; Singh et al., 2017). Estimation of ECM components divulged the ability of UMB to reduce proteins, lipids, carbohydrates and eDNA content (Figure 6). This result was corroborated by FTIR analysis of EPS, which divulged the overall reduction of biopolymers upon UMB treatment (Figure 7). Altogether, these results suggest the positive role of UMB in antibiotic susceptibility. Thus, in continuation, AST was performed in the absence and presence of UMB. As anticipated, UMB embellished the antibacterial activity of various classes of tested antibiotics (Table 3). Hence, UMB could be used in clinical settings to improvise the antibacterial activity of antibiotics.

Gene expression analysis was performed to unravel the plausible mechanism underlying the antibiofilm potential of UMB. The qPCR results divulged the significant down regulation of important genes involved in biofilm formation and virulence factors production (Figure 8). UMB down regulated *agrA* (response regulator of agr quorum sensing system), which in turn, indicates reduction of virulence, invasiveness and resistance to host defense molecules and antibiotic treatments. However, an earlier study with agr mutants recorded thicker biofilm formation than agr wild type owing to the increased expression of *atlE* (bifunctional autolysin/adhesin) (Dai et al., 2012). Conversely, UMB down regulated *atlE* expression in parallel to *agrA*. Nevertheless, this observation was found to be in line with Yao et al. (2006), who observed positive regulation of *atlE* in agr wild type. Also, Greenberg et al. (2018) have noticed significant down regulation of *atlE* in MRSE upon treatment with an AgrA inhibitor F19 (biaryl hydroxyketones). These contradictory observations in *atlE* expression could be attributed to varied level of *atlE* transcription during various phases of bacterial growth cycle (Vuong et al., 2003; Batzilla et al., 2006; Mack et al., 2007).

Additionally, *S. epidermidis* harbors several adhesin molecules known as microbial surface components recognizing adhesive matrix molecules (MSCRAMM) to facilitate binding of cells to matrix proteins (Bowden et al., 2005; Otto, 2009). UMB down regulated MSCRAMM encoding genes such as *atlE, sdrG, sdrF*, and *ebh*, which signifies the hindrance in bacterial attachment to host matrix proteins. UMB also down regulated the genes encoding adhesive molecules such as *icaD* (ica dependent biofilm formation), *aap* and *bhp* (ica independent biofilm formation). The up regulation of *icaA* upon UMB treatment is envisaged to result in truncated PIA, as transferase activity of *icaA* product becomes substantial for oligomer synthesis longer than 20 residues only when co-expressed with *icaD* product (Arciola et al., 2015). Thus, qPCR result suggests that, UMB hampers biofilm and virulence of MRSE by down regulating the genes encoding adhesive molecules and initial attachment.

*In vivo* studies were performed in *C. elegans*, which simulates copious cellular functions and disease related gene orthologs of humans (Hunt, 2017). The cytotoxicity assay divulged the non-toxic nature and health benefits of UMB since survival of UMB fed group was slightly higher than *E. coli* OP50 fed group. This result was in pact with a previous study wherein, the body weight of experimental rats fed daily with 30 mg/kg body weight of UMB for 30 weeks was witnessed to be increased (Muthu et al., 2013). UMB partially improved the survivability of MRSE infected *C. elegans*, which was also corroborated by CFU assay (Figures 9A,B), further signifying the antivirulence ability of UMB.

Furthermore, the ability of UMB to reduce bacterial adherence to type I Cn was confirmed using *C. elegans*, as its cuticle is a Cn-rich layer built up with over 154 genes encoding for Cn (Johnstone, 2000). Atkinson et al. (2011) have observed that *Yersinia pseudotuberculosis*, a Gram negative pathogenic bacterium establishes matrix-encased multilayered biofilm on live surface of *C. elegans* cuticle by colonizing its anterior part. Besides, *Yersinia* spp. produce polymeric N-acetyl-D-glucosamine like polysaccharide found in *Staphylococcus* spp. for establishing biofilm (Otto, 2009). To the best of the investigators' knowledge, the present study is the first report to analyze biofilm formation of *S. epidermidis*, a Gram positive bacterium on live surface of *C. elegans* cuticle. UMB reduced MRSE adherence to *C. elegans* cuticle which was contrasted with untreated MRSE that formed multilayered biofilm on anterior part of *C. elegans* (Figure 9C) similar to *Y. pseudotuberculosis*. This observation agrees with *in vitro* Cn binding assay and gene expression analysis, wherein UMB treatment decreased bacterial adherence to Cn coated surface and down regulated the expression of *sdrF* gene essential for Cn binding, respectively. Overall, the *in vivo* assays unearthed the antibiofilm and antivirulence potential of UMB and its appropriateness for clinical applications.

## Conclusion

In conclusion, the present study demonstrates the antibiofilm and antivirulence property of UMB against *S. epidermidis*. The results revealed the competence of UMB to impede biofilm formation on polystyrene, glass and titanium surfaces via inhibition of initial attachment and intercellular adhesion. Consistent with this, UMB was found to decrease rate of aggregation and adherence to polystyrene and type-I Cn coated surfaces. Besides, UMB reduced production of slime and secreted hydrolases, which are crucial for establishment of biofilm and invasive lifestyle of *S. epidermidis*. The EPS content of biofilm was also found to be reduced upon UMB treatment, which is presumed to be the key source for enhanced antibiotic susceptibility of UMB treated MRSE. In this study, we hypothesize that UMB reduces biofilm formation and pathogenicity of MRSE by down regulating the genes crucial for initial attachment, intercellular adhesion, accumulation, and adherence to ECM proteins, as evidenced by *in vitro* assays. Further, the cytotoxicity assay in *C. elegans* revealed the innocuous nature of UMB. The *in vivo* virulence level, infection rate, and adherence of MRSE were greatly reduced upon UMB treatment, which signifies the application of UMB to treat *S. epidermidis* infections. Nonetheless, the suitability of UMB needs to be corroborated using higher eukaryotic models before advancing UMB to clinical applications.

## Data Availability Statement

All datasets generated for this study are included in the manuscript/supplementary files.

## Author Contributions

SP, TS, KB, GS, and MP designed the experiments. TS, MP, and VD performed the experiments. TS analyzed the data. TS, GS, and SP wrote the manuscript. All authors ratified the final manuscript.

### Conflict of Interest

The authors declare that the research was conducted in the absence of any commercial or financial relationships that could be construed as a potential conflict of interest.

## Abbreviations

UMB: umbelliferone
MRSE: methicillin-resistant *Staphylococcus epidermidis*
PIA: polysaccharide intercellular adhesin
Cn: collagen
CFCS: cell free culture supernatant
ECM: extracellular matrix
EPS: extracellular polymeric substances
MSCRAMM: microbial surface components recognizing adhesive matrix molecules
MBIC: minimum biofilm inhibitory concentration
AB: alamar blue
CV: crystal violet
OD: optical density
ANOVA: analysis of variance.
